# Demographic and clinical characteristics, seizure disorders, and antiepileptic drug usage in different types of corpus callosum disorders: a comparative study in children

**DOI:** 10.1186/s13052-024-01589-x

**Published:** 2024-01-25

**Authors:** Ru-Huei Fu, Po-Yen Wu, I-Ching Chou, Chien-Heng Lin, Syuan-Yu Hong

**Affiliations:** 1 Graduate Institute of Biomedical Sciences, China Medical University, 40402 Taichung, Taiwan; 2 Translational Medicine Research Center, China Medical University Hospital, 40447 Taichung, Taiwan; 3 Division of Pediatric Neurology, China Medical University Children’s Hospital, 2 Yuh-Der Road, 40447 Taichung, Taiwan; 4 College of Chinese Medicine, Graduate Institute of Integrated Medicine, China Medical University, 40402 Taichung, Taiwan; 5 Division of Pediatrics Pulmonology, China Medical University Children’s Hospital, 40447 Taichung, Taiwan; 6 Department of Biomedical Imaging and Radiological Science, College of Medicine, China Medical University, 40402 Taichung, Taiwan; 7 Department of Medicine, School of Medicine, China Medical University, 40402 Taichung, Taiwan

**Keywords:** Corpus callosum disorders (CCD), Seizure disorders, Antiepileptic drugs (AEDs), Demographic characteristics, Clinical characteristics

## Abstract

**Background:**

This study aimed to investigate the demographic and clinical characteristics, types of seizure disorders, and antiepileptic drug usage among individuals with different types of corpus callosum disorders.

**Methods:**

A total of 73 individuals were included in the study and divided into three groups based on the type of corpus callosum abnormality: hypoplasia (H), agenesis (A), and dysgenesis (D). Demographic data, including gender and preterm birth, as well as clinical characteristics such as seizure disorders, attention deficit hyperactivity disorder (ADHD), severe developmental delay/intellectual disability, and other brain malformations, were analyzed. The types of seizure disorders and antiepileptic drugs used were also examined.

**Results:**

The H group had the highest number of participants (*n* = 47), followed by the A group (*n* = 11) and the D group (*n* = 15). The A group had the highest percentage of males and preterm births, while the D group had the highest percentage of seizure disorders, other brain malformations, and severe developmental delay/intellectual disability. The A group also had the highest percentage of ADHD. Focal seizures were observed in all three groups, with the highest proportion in the A group. Focal impaired awareness seizures (FIAS) were present in all groups, with the highest proportion in the D group. Generalized tonic-clonic seizures (GTCS) were observed in all groups, with the highest proportion in the H group. Different types of antiepileptic drugs were used among the groups, with variations in usage rates for each drug.

**Conclusion:**

This study provided insights into the demographic and clinical characteristics, seizure disorders, and antiepileptic drug usage among individuals with different types of corpus callosum disorders. Significant differences were found between the groups, indicating the need for tailored management approaches. However, the study has limitations, including a small sample size and a cross-sectional design. Further research with larger sample sizes and longitudinal designs is warranted to validate these findings and explore the relationship between corpus callosum abnormality severity and clinical outcomes.

**Supplementary Information:**

The online version contains supplementary material available at 10.1186/s13052-024-01589-x.

## Introduction

Corpus callosum disorders (CCD) encompass a range of neurological and developmental issues resulting from abnormalities in the development of the corpus callosum, a crucial brain structure responsible for interhemispheric communication [1, 2]. These disorders can lead to various clinical manifestations, including seizure disorders and cognitive impairments [3]. Understanding the demographic and clinical characteristics of individuals with different types of corpus callosum disorders is essential for improving diagnostic accuracy and guiding treatment strategies [4, 5].

Previous studies have shed light on the prevalence and etiology of corpus callosum disorders. For instance, a study reported that the overall incidence of corpus callosum abnormalities was estimated to be between 1.8 per 10,000 livebirths to 230–600 per 10,000 in children [6]. Moreover, genetic and environmental factors, including prenatal exposure to teratogenic agents, have been linked to corpus callosum abnormalities [7–9].

While previous studies have explored corpus callosum disorders, there remains a need for comprehensive investigations comparing demographic and clinical features, types of seizure disorders, and antiepileptic drug usage among different types of corpus callosum abnormalities. Such research can contribute to a better understanding of the varied presentations and inform personalized management approaches.

Seizure disorders represent one of the common clinical features associated with corpus callosum disorders. According to a study by Unterberger et al. [10], two thirds of individuals with agenesis of the corpus callosum experienced seizures. Seizures in corpus callosum disorders can manifest as different types, including generalized tonic-clonic seizures (GTCS), focal impaired awareness seizures (FIAS), focal aware seizures (FAS), myoclonic seizures, atonic seizures, and absence seizures. Characterizing the types of seizures associated with different types of corpus callosum disorders is crucial for accurate diagnosis and appropriate management [5].

Furthermore, the choice and usage of antiepileptic drugs (AEDs) play a vital role in the management of seizures in individuals with corpus callosum disorders. However, there is limited research investigating the specific AED usage patterns in this population. Understanding the types of AEDs used and their effectiveness can aid clinicians in optimizing treatment regimens and minimizing adverse effects [11].

This study aims to address these gaps by examining the demographic and clinical characteristics, types of seizure disorders, and antiepileptic drug usage among individuals with hypoplasia, agenesis, and dysgenesis of the corpus callosum. By comparing and analyzing these factors, the study aims to provide valuable insights into the clinical implications of corpus callosum abnormalities and aid clinicians in tailoring their management strategies accordingly.

## Material and methods

In this retrospective cohort study, we aimed to explore the demographic and clinical characteristics of patients with various types of CCD, as well as their associated seizure patterns and the utilization of antiepileptic drugs. We employed a rigorous diagnostic basis and a comprehensive array of diagnostic methods to categorize seizure patterns in these individuals.

### Study cohort selection

The subjects were categorized into three distinct groups, following the diagnostic criteria and classification of CCD as outlined by [12]:

1. Group H (Hypoplasia of the Corpus Callosum): Consisted of 47 individuals.
2. Group A (Agenesis of the Corpus Callosum): Consisted of 11 individuals.
3. Group D (Dysgenesis of the Corpus Callosum): Consisted of 15 individuals.

### Ethical approval and data collection

This study received approval from the Institutional Review Board of China Medical University Children’s Hospital (CMUH110-REC1-029(AR-1)). To assemble our study cohort, we conducted a comprehensive search of brain MRI records from a database containing imaging data of 300,000 children under the age of 18. The search spanned from January 1, 2012, to December 31, 2019. We subsequently followed these patients’ medical records until December 31, 2022, to identify individuals with confirmed diagnoses of CCD.

### Exclusions were made for patients who either

1. Deceased or were lost to follow-up during the tracking period.
2. Presented with unclear CCD diagnoses.
3. Possessed incomplete medical information, including seizure disorders, in the absence of accompanying EEG examinations or antiepileptic drug medication records.

**Fig. 1:**
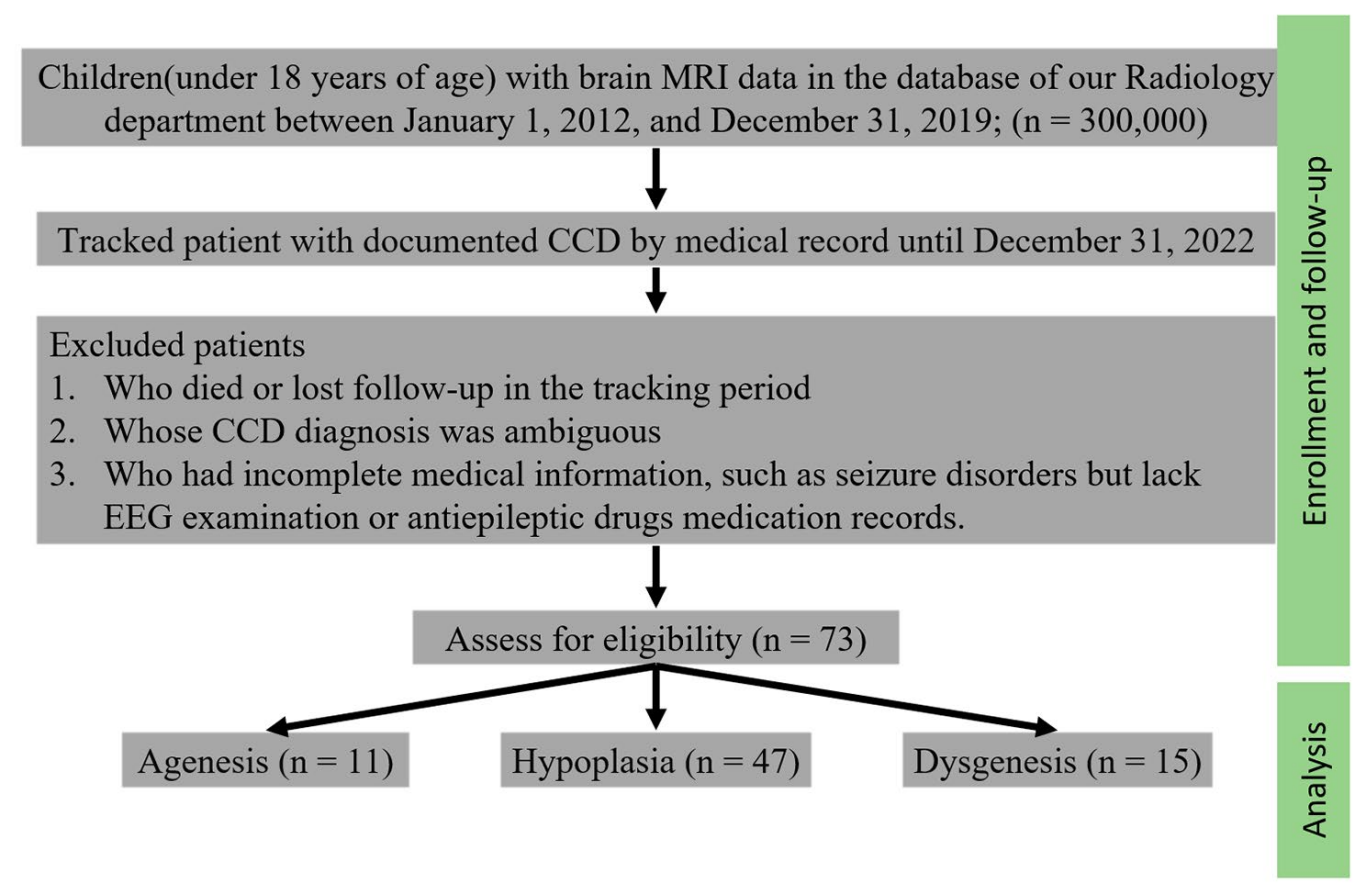
Flowchart of the study. *The patients who were screened from the medical records

Subsequently, we performed a thorough manual review of the remaining 73 children’s medical records to extract pertinent data related to their clinical characteristics, seizure patterns, and antiepileptic drug utilization (refer to Fig. 1).

### Seizure pattern classification

To classify and characterize seizure patterns, we adopted a standardized diagnostic approach. All patients underwent EEG examinations, which played a central role in identifying and distinguishing various seizure types, such as focal, generalized, or atypical seizures, within our study cohort. The EEG recordings were analyzed to identify potential triggers or predisposing factors for seizures. This included looking for patterns in the EEG data that might suggest certain triggers, such as specific activities, stress, or sleep-related events. We also examined the duration and frequency of seizures. This information is crucial in understanding the impact of seizures on the patients’ daily lives and in determining appropriate treatment strategies.

### Statistical analysis

Statistical analyses were conducted to assess differences among the groups. Continuous variables were compared using the Wilcoxon rank-sum test, and the results were reported as medians and interquartile ranges (IQR). Categorical variables were analyzed using the chi-squared test or Fisher’s exact test, with results presented as frequencies and percentages (%). All analyses were performed using SAS version 9.4 (SAS Institute Inc., Cary, NC) and were conducted in a 2-sided manner with a significance level set at 0.05.

## Results

Table 1 provides an overview of the demographic and clinical characteristics of individuals presenting with various types of corpus callosum disorders, namely agenesis (A), hypoplasia (H), and dysgenesis (D). The respective groups consisted of 11, 47, and 15 subjects. The variables considered encompassed gender, preterm birth, seizure disorders, attention deficit hyperactivity disorder (ADHD), severe developmental delay/intellectual disability, other brain malformations, polytherapy for seizures, and age. Notably, there are no significant differences among the three groups of subjects in terms of gender, birth, epilepsy, ADHD, developmental delay/mental retardation and other characteristics. The only significant difference was age, where the mean ages of groups A and D were significantly different, with the mean age of group D being higher than that of group A. In addition, other brain abnormalities and the number of people who need to use 2 or more epilepsy drugs to control the disease had near-significant differences between groups H and D, with a higher proportion in group D than in group H.

**Table 1:** Demographic and clinical characteristics of subjects with different types of corpus callosum disorders

Characteristic	A (*n* = 11)	H (*n* = 47)	D(*n* = 15)	A vs. H^*^	A vs. D^*^	H vs. D^*^
Sex (M)	8 (72.7%)	29 (61.7%)	7 (46.7%)	χ²=0.43, *p* = 0.51	χ²=2.24, *p* = 0.13	χ²=1.03, *p* = 0.31
Premature/Full-term infants (P/F)	5/6 (45.5%/54.5%)	12/35 (25.5%/74.5%)	6/9 (40%/60%)	χ²=1.67, *p* = 0.20	χ²=0.02, *p* = 0.88	χ²=0.93, *p* = 0.34
Developing epilepsy	7 (63.6%)	19 (40.4%)	9 (60%)	χ²=2.07, *p* = 0.15	χ²=0.06, *p* = 0.81	χ²=2.00, *p* = 0.16
Developing ADHD	3 (27.3%)	7 (14.9%)	1 (6.7%)	χ²=1.28, *p* = 0.26	χ²=2.29, *p* = 0.13	χ²=1.32, *p* = 0.25
Developing developmental delay/intelligence deficiency	8 (72.7%)	32 (68.1%)	12 (80%)	χ²=0.09, *p* = 0.77	χ²=0.23, *p* = 0.63	χ²=0.59, *p* = 0.44
Combined with other brain abnormalities	9 (81.8%)	35 (74.5%)	15 (100%)	χ²=0.29, *p* = 0.59	Fisher’s exact test, *p* = 0.10	Fisher’s exact test, *p* = 0.05
The number of people who need more than two epilepsy drugs	4 (57.1%)^†^	10(52.6%)^†^	8(88%)^†^	Fisher’s exact test,*p* = 0 0.75	Fisher’s exact test,*p* = 0 0.14	χ² =3 0.48,*p* = 0 0.06
Mean ± standard deviation of age (year)	7 0.3 ± 3 0.8	9 0.2 ± 5 0.1	11 0.4 ± 5 0.0	t=-1 0.38,*p* = 0 0.17	t=-2 0.30,*p* = 0 0.03	t=-1 0.58,*p* = 0 0.12

^*^χ², chi-square test; t, independent samples t-test

^†^Only subjects with epilepsy are counted here

Turning our attention to Table 2, it provides a comprehensive overview of the types of seizure disorders observed in individuals with different corpus callosum disorders. The number of subjects with seizure disorders in each group: Group A comprised *n* = 7 participants, constituting 63.6% of the total; Group H encompassed *n* = 19 individuals, representing 40.4%; and Group D included *n* = 9 subjects, comprising 60% of the cohort, respectively. The seizure disorder categories included FAS, FIAS, GTCS, and unclassified seizures. Interestingly, focal seizures were observed in all three groups, with the highest proportion recorded in Group A (85.8%). Additionally, FIAS was present in all three groups, with the highest proportion identified in Group D (66.7%). Furthermore, GTCS were prevalent across all three groups, with the highest proportion seen in Group H (26.3%). In terms of specific seizure disorder percentages within each group, the D group exhibited the highest proportion of FIAS, followed by the H group and then the A group. Conversely, the A group had the highest percentage of FAS, followed by the H group and then the D group. Lastly, the H group exhibited the highest proportion of unclassified seizures, followed by the A group and then the D group. It is noteworthy that no statistically significant differences were found between the groups for any seizure disorder type (*p* > 0.05 for all seizure disorder types; Fisher’s exact test).

**Table 2:** Types of seizure disorders among subjects with different types of corpus callosum disorders

Group	Number of subjects with seizure disorders	Focal aware seizures (FAS) (%)^†^	Focal impaired awareness seizures (FIAS) (%)^†^	Generalized tonic-clonic seizures (GTCS) (%)^†^	Unclassified seizures (%)^†^
A (Agenesis)	7 (63.6%)	3 (42.9)	3 (42.9)	1 (14.3)	1 (14.3)
H (Hypoplasia)	19 (40.4%)	4 (21.1)	9 (47.4)	5 (26.3)	6 (31.6)
D (Dysgenesis)	9 (60%)	1 (11.1)	6 (66.7)	1 (11.1)	1 (11.1)

^†^ The *p* value can be obtained by using a chi-square calculator or a chi-square table with df = 6 and χ2 = 4 0.64. The difference between groups was not statistically significant for any type of seizure disorder ^†^ (*p* > 0.05 for all types of seizure disorder; Fisher’s exact test)

Table 3 provides insights into the interictal EEG findings observed in subjects with varying types of corpus callosum disorders. The interictal EEG findings included frontal, temporal, central, occipital, generalized, multifocal, normal, and frontal to generalized patterns. Remarkably, the D group exhibited the highest percentage of multifocal and temporal patterns, followed by the H group and then the A group. Moreover, the H group displayed the highest proportion of frontal, central, occipital, generalized, normal, and frontal to generalized patterns, followed by the A group and then the D group. Notably, the A group did not exhibit any frontal or frontal to generalized patterns. Importantly, no statistically significant differences were found between the groups for any interictal EEG finding (*p* > 0.05 for all interictal EEG findings; Fisher’s exact test).

**Table 3:** Interictal EEG findings among subjects with different types of corpus callosum disorders

Group	Number of subjects with seizure disorders	Frontal (F) (%)^†^	Temporal (T) (%)^†^	Central (C) (%)^†^	Occipital (O) (%)^†^	Generalized (G) (%)^†^	Multifocal (%)^†^	Normal (N) (%)^†^	Frontal to generalized (F to G) (%)^†^
A (Agenesis)	7 (63.6%)	0 (0.0)	1 (14.3)	1 (14.3)	1 (14.3)	1 (14.3)	3 (42.9)	1 (14.3)	0 (0.0)
H (Hypoplasia)	19 (40.4%)	2 (10.5)	2 (10.5)	1 (5.3)	2 (10.5)	2 (10.5)	8 (42.1)	3 (15.8)	3 (15.8)
D (Dysgenesis)	9 (60%)	0 (0.0)	2 (22.2)	0 (0.0)	0 (0.0)	0 (0.0)	6 (66.7)	0 (0.0)	1 (11.1)

^†^ The difference between groups was not statistically significant for any interictal EEG finding (*p* > 0.05 for all interictal EEG findings; Fisher’s exact test)

Moving on to Table 4, the participants were categorized into three groups based on the type of corpus callosum abnormality. The table illustrates the number and percentage of patients in each group who developed seizure disorders, along with the types of AEDs administered. The AEDs were classified as VPA (valproic acid), LEV (levetiracetam), TPX (topiramate), OXC (oxcarbazepine), PB (phenobarbital), CLN (clonazepam), LMT (lamotrigine), LCM (lacosamide), VGB (vigabatrin), PRG (pregabalin), and CLB (clobazam). Notably, the results indicated significant variations among the groups in terms of the AEDs utilized. Specifically, the D group exhibited the highest percentage of patients using VPA, TPX, VGB, PRG, and CLB, while the H group had the highest percentage of patients using LEV, OXC, PB, CLN, and LMT. The A group, on the other hand, had the lowest percentage of patients using any antiepileptic drug, except for FAS and LCM, which were similar to the H group. It is essential to explore potential explanations for these findings. Different types of corpus callosum abnormalities may exert varying effects on seizure patterns and responses to antiepileptic drugs.

**Table 4:** Types of AEDs used by patients with different types of corpus callosum abnormalities

Group	Number of subjects	Number of subjects with seizure disorders	Percentage of subjects with seizure disorders	AEDs used (number of subjects)	***p***-value for seizure disorders	***p***-value for AEDs	Test method
A	11	7	63.6%	VPA (3), LEV (3), TPX (1), OXC (2), CLN (1), LCM (1), CLB (1)	0.02 vs. H^*^, 0.34 vs. D	0.04 vs. H^*^, 0.12 vs. D	Chi-square
H	47	19	40.4%	VPA (6), LEV (11), TPX (1), OXC (5), PB (4), CLN (7), LMT (2), LCM (1)	0.02 vs. A^*^, 0.09 vs. D	0.04 vs. A^*^, 0.07 vs. D	Chi-square
D	15	9	60.0%	VPA (7), LEV (3), TPX (3), OXC (1), CLN (3), LCM (1), VGB (3), PRG (1), CLB (1)	0.34 vs. A, 0.09 vs. H	0.12 vs. A, 0.07 vs. H	Chi-square

^*^p < 0.05 indicates significant differences among the groups in terms of the types of AEDs they used

Finally, in Table 5, the study undertook a comprehensive comparison of the clinical features across three groups of subjects with diverse corpus callosum disorders. The respective group sizes were 11, 47, and 15. The prevalence of seizure disorders and malformations of cortical development (MCD) was calculated for each group, along with the types of MCD. Notably, the D group exhibited the highest percentage of both seizure disorders and MCD, followed by the A group and then the H group. Statistically significant differences were found between the groups regarding MCD (*p* < 0.05, chi-square test), while no significant differences were observed for seizure disorders (*p* > 0.05, Fisher’s exact test). Furthermore, the types of MCD varied among the groups, with colpocephaly being the most prevalent in the A group, schizencephaly in the H group, and macrogyria/pachygyria in the D group. These findings underscore the distinctive clinical implications associated with different types of corpus callosum disorders, potentially suggesting diverse underlying mechanisms.

**Table 5:** Comparison of clinical features among different types of corpus callosum disorders

Group	Number of subjects	Seizure disorders (%)^*^	Malformations of cortical development (MCD) (%)^†^	Types of MCD
A (Agenesis)	11	7 (63.6)	6 (54.5)	Heterotopia (1), polymicrogyria (1), colpocephaly (3), schizencephaly (1), holoprosencephaly (1), porencephaly (1)
H (Hypoplasia)	47	19 (40.4)	5 (10.6)	Focal cortical dysplasia (1), heterotopia (1), pachygyria (1), polygmicrogyria (1), schizencephaly (2)
D (Dysgenesis)	15	9 (60.0)	9 (60.0)	Focal cortical dysplasia (2), holoprosencephaly (1), macrogyria/pachygyria (3), porencephaly (2), schizencephaly (1), heterotopia (1), polygmicrogyria(1)

^*†^ The difference between groups was statistically significant for MCD ^†^ (*p* < 0.05, chi-square test), but not for seizure disorders^*^ (*p* > 0.05, Fisher’s exact test)

The study also examined the correlation between MCD and seizure frequency in individuals with various corpus callosum disorders. The results showed that the D group had the highest percentage of both seizure disorders (*n* = 9, 60%) and MCD (*n* = 9, 60%), followed by the A group (*n* = 7, 63.6%; *n* = 6, 54.5%)and then the H group (*n* = 19, 40.4%; *n* = 5, 10.6%). Statistically significant differences were observed for MCD between the groups (*p* < 0.05, chi-square test), indicating that the type of corpus callosum abnormality is associated with different patterns of MCD. However, no statistically significant differences were found for seizure disorders (*p* > 0.05, Fisher’s exact test), suggesting that the presence of MCD might not significantly influence seizure frequency across these groups.

## Discussion

The current study delved into a comprehensive exploration of the demographic and clinical features, the types of seizure disorders, and the usage of AEDs in individuals with various CCD. The corpus callosum, a vital brain structure responsible for facilitating interhemispheric communication, plays a crucial role in neurodevelopment. Anomalies in its development can lead to a wide spectrum of neurological and developmental issues [8, 13, 14]. Our study cohort consisted of 73 participants who were divided into three groups based on the type of CCD: agenesis (A), hypoplasia (H), and dysgenesis (D).

### Relationship between clinical characteristics and CCD-related epilepsy

Our findings offer valuable insights into the intricate relationship between clinical characteristics and epilepsy associated with CCD. Specifically, we observed that among children in groups D, A, and H, group D exhibited the highest prevalence of seizure disorders, other brain malformations, and the requirement for multiple medications to manage epilepsy [15, 16]. This observation may be attributed to the more severe abnormalities associated with dysgenesis. Dysgenesis of the corpus callosum, a structure responsible for connecting the brain’s hemispheres, has the potential to disrupt communication between them, potentially elevating the risk of epileptic seizures and other brain malformations [17, 18]. Existing research has also suggested a correlation between corpus callosum abnormalities and an increased predisposition to epilepsy and other neurological disorders [17–19]. The corpus callosum is well-recognized for its fundamental role in facilitating communication between both brain hemispheres, and any aberrations in its development can result in cognitive and developmental challenges [20, 21].

Moreover, our study revealed that group A displayed the highest proportion of children diagnosed with ADHD, followed by group H, and then group D. Although we do not currently possess a complete understanding of the underlying pathology of this result, it is plausible that it may be connected to the corpus callosum and its role in regulating interactions and coordination among different brain regions [22–24].

### Types of seizure disorders and AEDs usage

With regard to the types of seizure disorders, FAS were observed among all three groups, with the highest prevalence identified in Group A (42.9%). Our study did not identify statistically significant differences between groups for any type of seizure disorder. Nonetheless, this observation suggests that individuals with different types of CCD may necessitate personalized approaches to AEDs selection [25, 26]. While our study does not offer specific recommendations for AEDs selection in patients with epilepsy and CCD, it suggests that individuals with different types of CCD may require individualized approaches to AEDs choice [27, 28].

### Distinctive prevalence of MCD, diverse clinical implications of different CCD types and the correlation between MCD and seizure frequency

One of the key findings is the statistically significant differences identified among the groups regarding the presence of MCD (*p* < 0.05, chi-square test). This observation highlights that the type of corpus callosum abnormality is associated with varying patterns of MCD. In essence, the type of CCD plays a crucial role in shaping the profile of associated MCD. Specifically, colpocephaly was found to be the most prevalent type of MCD in the A group, schizencephaly in the H group, and macrogyria/pachygyria in the D group.

These findings underscore the distinctive clinical implications associated with different types of corpus callosum disorders. It suggests that there might be diverse underlying mechanisms contributing to the development of MCD in individuals with CCD [29, 30]. While further research is needed to elucidate the precise mechanisms involved, these findings emphasize the need for personalized assessments and tailored approaches in the clinical management of individuals with CCD.

The study also examined the correlation between the presence of MCD and seizure frequency in individuals with various types of corpus callosum disorders. It is noteworthy that while the D group exhibited the highest percentage of both seizure disorders and MCD, statistically significant differences were observed only for MCD (*p* < 0.05, chi-square test). This suggests that the type of corpus callosum abnormality appears to be associated with different patterns of MCD, potentially influencing the specific MCD types seen in each group.

However, no statistically significant differences were found for seizure disorders (*p* > 0.05, Fisher’s exact test). This implies that, while MCD is associated with the type of CCD, it might not significantly influence the frequency of seizures across these groups. This highlights the complexity of the relationship between MCD, CCD, and epilepsy. It is important to consider that epilepsy is a multifactorial condition, and the mere presence of MCD may not be the sole determinant of seizure frequency in individuals with CCD.

### Implications and future directions

Effectively managing epilepsy in individuals with CCD necessitates a multidisciplinary approach, involving neurology, neuroimaging, genetics, and neuropsychology. Given the diversity of corpus callosum abnormalities and their associated clinical manifestations, a personalized approach to management is essential. This may encompass:

1. Precise Genetic Testing: Identifying underlying genetic causes to inform treatment strategies.
2. Advanced Neuroimaging Techniques: Evaluating the severity and extent of corpus callosum abnormalities.
3. Comprehensive Neuropsychological Assessments: Guiding individualized intervention plans.

Collaborative efforts among specialists from different disciplines will advance our understanding of the intricate relationship between corpus callosum abnormalities, epilepsy, and neurodevelopmental outcomes, ultimately leading to more targeted and effective treatment strategies [6, 8, 30].

### Epilepsy surgery and corpus callosectomy

This study also contributes valuable insights into the potential role of epilepsy surgery, specifically corpus callosectomy, in individuals with epilepsy and corpus callosum disorders. The observed higher prevalence of focal awareness seizures, focal impaired awareness seizures, and generalized tonic-clonic seizures in all three groups (A, H, and D) suggests that these individuals may experience refractory or difficult-to-control seizures.

Corpus callosectomy, involving the surgical resection or disconnection of the corpus callosum, has been proposed as a therapeutic approach for medically intractable epilepsy associated with corpus callosum abnormalities [31, 32]. The procedure aims to prevent the spread of epileptic activity between hemispheres, potentially reducing the frequency and severity of seizures [33]. Although the study does not specifically indicate a preference for CCD selection in epilepsy surgery, the information presented suggests that dysgenesis (group D) may be a favorable CCD type for epilepsy surgery due to its higher rates of seizure disorders and other brain malformations. Dysgenesis often involves more severe structural abnormalities of the corpus callosum, which may increase the likelihood of interhemispheric propagation of epileptic activity [34]. Disconnecting the corpus callosum in cases of dysgenesis can potentially reduce the spread of seizures [15, 35].

It is important to note that the decision to pursue epilepsy surgery, including corpus callosectomy, is complex and requires a comprehensive evaluation of each individual’s specific case. Factors such as the type and frequency of seizures, associated comorbidities, neuroimaging findings, and the overall impact on quality of life need to be carefully considered [36]. The involvement of a multidisciplinary team, including neurologists, neurosurgeons, neuropsychologists, and neuroradiologists, is crucial in determining the appropriateness and potential benefits of epilepsy surgery for patients with epilepsy and CCD [37, 38]. Further research is needed to assess the long-term outcomes and effectiveness of corpus callosectomy in reducing seizures and improving neurodevelopmental outcomes in this population. Prospective studies with larger sample sizes and longitudinal follow-up can provide more definitive evidence regarding the efficacy, safety, and patient selection criteria for epilepsy surgery, including corpus callosectomy, in individuals with epilepsy and CCD.

### Limitations

It is important to acknowledge the limitations of this study. The relatively small sample size, particularly for the dysgenesis group, may limit the generalizability of the findings to larger populations. Secondly, the study adopted a cross-sectional design, which restricts the ability to establish causality or determine the temporal relationships.

## Conclusions

This study offers valuable insights into the demographic and clinical characteristics, seizure disorder types, and use of AEDs among individuals with various CCD. Notably, significant differences in these variables were observed among the three groups: corpus callosum dysgenesis, agenesis, and hypoplasia. These findings emphasize the need for tailored evaluation and management strategies based on the specific CCD subtype in epilepsy patients. Although no specific seizure type stood out statistically, patients with corpus callosum agenesis exhibited a higher percentage of ADHD, followed by hypoplasia and dysgenesis. Furthermore, the D group had the highest proportions of seizure disorders, other brain malformations and the need of polytherapy for seizures, followed by the A group and then the H group, although no group revealed any statistically significant in particular seizure type. Agenesis patients displayed highest percentage of ADHD followed by H group and D group. D group exhibited the highest percentage of both seizure disorders and MCD, followed by the A group and then the H group. At last, the study’s findings provide valuable insights into the intricate relationship between MCD and CCD. These findings emphasize the importance of considering the type of CCD when assessing the risk and types of associated MCD. Furthermore, while MCD may play a role in the clinical manifestation of CCD, it may not be the sole factor influencing the frequency of seizures. This underscores the need for comprehensive and individualized approaches in understanding and managing epilepsy in individuals with diverse corpus callosum disorders. Overall, these results contribute to our understanding of the clinical implications of corpus callosum abnormalities and aid clinicians in optimizing patient care.

### Electronic supplementary material

Below is the link to the electronic supplementary material.

Supplementary Material 1

Supplementary Material 2

Supplementary Material 3

## Data Availability

The datasets used and/or analyzed during the current study are available from the corresponding author on reasonable request.

## Abbreviations

AEDs: Antiepileptic Drugs
ADHD: Attention Deficit Hyperactivity Disorder
ANOVA: Analysis of Variance
CCD: Corpus Callosum Disorders
CLB: Clobazam
CLN: Clonazepam
CMUH: China Medical University Children’s Hospital
EEG: Electroencephalogram
FAS: Focal Aware Seizures
FIAS: Focal Impaired Awareness Seizures
GTCS: Generalized Tonic-Clonic Seizures
IQR: Interquartile Ranges
LCM: Lacosamide
LEV: Levetiracetam
LMT: Lamotrigine
MCD: Malformations of Cortical Development
MRI: Magnetic Resonance Imaging
OXC: Oxcarbazepine
PB: Phenobarbital
PRG: Pregabalin
SAS: Statistical Analysis System
TPX: Topiramate
VGB: Vigabatrin
VPA: Valproic Acid

## References

[1] Bernardes da Cunha S, Carneiro MC, Miguel Sa M, Rodrigues A, Pina C (2021). Neurodevelopmental outcomes following prenatal diagnosis of isolated Corpus Callosum Agenesis: a systematic review. Fetal Diagn Ther.

[2] Edwards TJ, Fenlon LR, Dean RJ (2020). Altered structural connectivity networks in a mouse model of complete and partial dysgenesis of the corpus callosum. NeuroImage.

[3] Huang X, Du X, Song H (2015). Cognitive impairments associated with corpus callosum infarction: a ten cases study. Int J Clin Exp Med.

[4] Szabó N, Gergev G, Kóbor J, Bereg E, Túri S, Sztriha L (2011). Corpus callosum anomalies: birth prevalence and clinical spectrum in Hungary. Pediatr Neurol.

[5] Hofman J, Hutny M, Sztuba K, Paprocka J (2020). Corpus Callosum Agenesis: an insight into the etiology and spectrum of symptoms. Brain Sci.

[6] Pânzaru MC, Popa S, Lupu A, Gavrilovici C, Lupu VV, Gorduza EV (2022). Genetic heterogeneity in corpus callosum agenesis. Front Genet.

[7] Pavone P, Falsaperla R, Ruggieri M, Praticò AD, Pavone L (2013). West syndrome treatment: new roads for an old syndrome. Front Neurol.

[8] Edwards TJ, Sherr EH, Barkovich AJ, Richards LJ (2014). Clinical, genetic and imaging findings identify new causes for corpus callosum development syndromes. Brain.

[9] Georgieff MK, Tran PV, Carlson ES (2018). Atypical fetal development: fetal alcohol syndrome, nutritional deprivation, teratogens, and risk for neurodevelopmental disorders and psychopathology. Dev Psychopathol.

[10] Unterberger I, Bauer R, Walser G, Bauer G (2016). Corpus callosum and epilepsies. Seizure.

[11] Kim KT, Kim DW, Yang KI (2020). Refining General principles of Antiepileptic Drug Treatments for Epilepsy. J Clin Neurol.

[12] Severino M, Geraldo AF, Utz N et al. Definitions and classification of malformations of cortical development: practical guidelines [published correction appears in Brain. 2020;143(12):e108]. *Brain*. 2020;143(10):2874–2894. 10.1093/brain/awaa174. 10.1093/brain/awaa174 PMC7586092 32779696

[13] Paul LK (2011). Developmental malformation of the corpus callosum: a review of typical callosal development and examples of developmental disorders with callosal involvement. J Neurodev Disord.

[14] Al-Hashim AH, Blaser S, Raybaud C, MacGregor D (2016). Corpus callosum abnormalities: neuroradiological and clinical correlations. Dev Med Child Neurol.

[15] Spencer SS (1988). Corpus callosum section and other disconnection procedures for medically intractable epilepsy. Epilepsia.

[16] Sutton VR, Van den Veyver IB, Adam MP, Mirzaa GM, Pagon RA (2006). Aicardi Syndrome. GeneReviews®.

[17] Kortüm F, Jamra RA, Alawi M (2018). Clinical and genetic spectrum of AMPD2-related pontocerebellar hypoplasia type 9. Eur J Hum Genet.

[18] Yuan J, Song X, Kuan E (2020). The structural basis for interhemispheric functional connectivity: evidence from individuals with agenesis of the corpus callosum. Neuroimage Clin.

[19] Roland JL, Snyder AZ, Hacker CD (2017). On the role of the corpus callosum in interhemispheric functional connectivity in humans. Proc Natl Acad Sci U S A.

[20] Hinkley LB, Marco EJ, Brown EG (2016). The contribution of the Corpus Callosum to Language lateralization. J Neurosci.

[21] Hall SA, Bell RP, Davis SW, Towe SL, Ikner TP, Meade CS (2021). Human immunodeficiency virus-related decreases in corpus callosal integrity and corresponding increases in functional connectivity. Hum Brain Mapp.

[22] Maxfield M, Cooper MS, Kavanagh A, Devine A, Gill Atkinson L. On the outside looking in: a phenomenological study of the lived experience of Australian adults with a disorder of the corpus callosum. *Orphanet J Rare Dis*. 2021;16(1):512. Published 2021 Dec 14. 10.1186/s13023-021-02140-5. 10.1186/s13023-021-02140-5 PMC8670101 34906174

[23] Martin LA, Hsu FW, Herd B, Gregg M, Sample H, Kaplan J (2021). Executive functions in agenesis of the corpus callosum: working memory and sustained attention in the BTBR inbred mouse strain. Brain Behav.

[24] Vasung L, Yun HJ, Feldman HA, Grant PE, Im K (2020). An atypical Sulcal Pattern in Children with disorders of the Corpus Callosum and its relation to behavioral outcomes. Cereb Cortex.

[25] Graham D, Tisdall MM, Gill D (2016). Corpus callosotomy outcomes in pediatric patients: a systematic review. Epilepsia.

[26] Rosenow F, van Alphen N, Becker A (2017). Personalized translational epilepsy research - novel approaches and future perspectives: part I: clinical and network analysis approaches. Epilepsy Behav.

[27] Azar NJ, Abou-Khalil BW (2008). Considerations in the choice of an antiepileptic drug in the treatment of epilepsy. Semin Neurol.

[28] Scheffner D (1980). Principles of anticonvulsant therapy in childhood epilepsies. Monogr Neural Sci.

[29] Velagapudi L, Matias CM, Ambrose TM (2021). Alternate seizure spread with Agenesis of the Corpus Callosum. J Epilepsy Res.

[30] Schell-Apacik CC, Wagner K, Bihler M (2008). Agenesis and dysgenesis of the corpus callosum: clinical, genetic and neuroimaging findings in a series of 41 patients. Am J Med Genet A.

[31] Okanishi T, Fujimoto A (2021). Corpus Callosotomy for Controlling Epileptic spasms: a proposal for Surgical selection. Brain Sci.

[32] Luat AF, Asano E, Kumar A, Chugani HT, Sood S (2017). Corpus Callosotomy for Intractable Epilepsy Revisited: the children’s hospital of Michigan Series. J Child Neurol.

[33] Itamura S, Okanishi T, Nishimura M (2019). Analysis for the Association between Corpus Callosum thickness and Corpus Callosotomy outcomes for patients with epileptic spasms or Tonic spasms. Pediatr Neurol.

[34] Lazarev VV, de Carvalho Monteiro M, Vianna-Barbosa R, deAzevedo LC, Lent R, Tovar-Moll F (2016). Electrophysiological correlates of Morphological Neuroplasticity in Human Callosal Dysgenesis. PLoS ONE.

[35] Kishima H, Oshino S, Tani N (2013). Which is the most appropriate disconnection surgery for refractory epilepsy in childhood?. Neurol Med Chir (Tokyo).

[36] Vaddiparti A, Huang R, Blihar D (2021). The evolution of Corpus Callosotomy for Epilepsy Management. World Neurosurg.

[37] Wieser HG (1996). Epilepsy surgery. Baillieres Clin Neurol.

[38] Rocque BG, Davis MC, McClugage SG (2018). Surgical treatment of epilepsy in Vietnam: program development and international collaboration. Neurosurg Focus.

