# Supplementary material for: Demographic and clinical characteristics, seizure disorders, and antiepileptic drug usage in different types of corpus callosum disorders: a comparative study in children
Source: Ital J Pediatr. 2024 Jan 25;50:20. doi: 10.1186/s13052-024-01589-x (PMC10809518; doi:10.1186/s13052-024-01589-x)
Supplement: Supplementary file 1 — Supplementary Material 1 [file 13052_2024_1589_MOESM1_ESM.pdf]

SPRINGER NATURE

Change of authorship request form - Journals (pre-acceptance)

Important information. Please read.

- This form should be used by authors to request any change in authorship (adding/deleting authors) including changes in corresponding authors. This form should not be used for name changes. Please fully complete all sections. Use black ink and block capitals and provide each author's full name with the given name first followed by the family name.

- By signing this declaration, all authors guarantee that the order of the authors are in accordance with their scientific contribution, if applicable as different conventions apply per discipline, and that only authors have been added who made a meaningful contribution to the work.

- Please note, in author collaborations where there is formal agreement for representing the collaboration, it is sufficient for the representative or legal guarantor (usually the corresponding author) to complete and sign the Authorship Change Form on behalf of all authors, **next to the added/removed author(s)**. **(Complete Section 3, followed by Section 6.)**
  In author collaborations where there is no formal agreement for representing the collaboration and **there are more than 10 authors**, one may sign for all, provided the signer appends correspondence that attests that each of the authors have agreed to the change **and the added/removed authors sign the form**. **(Complete Section 3, followed by Section 6.)**

- Please note, we cannot investigate or mediate any authorship disputes. If you are unable to obtain agreement from all authors (including those who you wish to be removed) you must refer the matter to your institution(s) for investigation. Please inform us if you need to do this.

- If you are not able to return a fully completed form within **30 days** of the date that it was sent to the author requesting the change, we may have to withdraw your manuscript. We cannot publish manuscripts where authorship has not been agreed by all authors (including those who have been removed).

- Incomplete forms will be rejected.

- Please return/upload this form, fully completed, to the Journals Editorial Office. The Journal and/or Publisher will consider the information you have provided to decide whether to approve the proposed change in authorship. We may decide to contact your institution for more information or undertake a further investigation, if appropriate, before making a final decision.

SPRINGER NATURE Springer Nature is one of the world's leading global research, educational and professional publishers, created in May 2015 through the combination of Nature Publishing Group, Palgrave Macmillan, Macmillan Education and Springer Science+Business Media.

SPRINGER NATURE

Change of authorship request form - Journals (pre-acceptance)

Section 1: Please provide the current title of manuscript

Manuscript ID no.: ITJP-D-23-00378

Title: Demographic and Clinical Characteristics, Seizure Disorders, and Antiepileptic Drug Usage in Different Types of Corpus Callosum Disorders: A Comparative Study in Children

Section 2: Please provide the previous authorship, in the order shown on the manuscript before the changes were introduced. Please indicate the corresponding author by adding (CA) behind the name.

	First name(s)	Family name	ORCID or SCOPUS id, if available
1st author	Bo-Yan	Wu	
2nd author	I-Ching	Chou	
3rd author	Chien-Heng	Lin	
4th author	Syuan-Yu	Hong	
5th author			
6th author			
7th author			
8th author			
9th author			
10th author			

Please use an additional sheet if there are more than 10 authors.

SPRINGER NATURE Springer Nature is one of the world's leading global research, educational and professional publishers, created in May 2015 through the combination of Nature Publishing Group, Palgrave Macmillan, Macmillan Education and Springer Science+Business Media.

SPRINGER NATURE

Change of authorship request form - Journals (pre-acceptance)

Section 3: Please provide a justification for change. Please use this section to explain your reasons for changing the authorship of your manuscript, e.g. what necessitated the change in authorship? Please refer to the (journal) policy pages for more information about authorship. Please explain why omitted authors were not originally included and/or why authors were removed on the submitted manuscript.

Dear Reviewers,

I would like to extend my heartfelt apologies for the oversights and errors in the submission of our manuscript. As the corresponding author, the responsibility for the accuracy and completeness of the submission ultimately rests with me, and I deeply regret the mistakes that were made. Specifically, I acknowledge that there was an unfortunate omission of the first author of this article, Ru-Huei Fu (RHF), and a significant error in the placement of another author with equal contribution, Po-Yen Wu (PYW), who was mistakenly identified as Bo-Yan Wu (BYW). These errors are entirely my responsibility, and I take full accountability for them. I wish to express my sincerest apologies to Ru-Huei Fu and Po-Yen Wu for any distress or inconvenience these errors may have caused. Both authors have made substantial contributions to this work, and their names and contributions should be accurately reflected. To rectify these issues, I am taking immediate steps to correct the order of the authors and ensure that all author names are represented correctly in the manuscript. The corrected version will accurately reflect the contributions of each author, including Ru-Huei Fu and Po-Yen Wu. I genuinely appreciate your understanding and patience as we work to address these issues promptly. Your feedback is essential to improving the quality of our research, and we are committed to delivering a revised manuscript that meets the highest standards of accuracy and integrity.

Once again, I apologize for any inconvenience these errors may have caused and appreciate your understanding in this matter.

Sincerely,
Dr. Syuan-Yu Hong (Corresponding Author)

Section 4: Proposed new authorship. Please provide your new authorship list in the order you would like it to appear on the manuscript. Please indicate the corresponding author by adding (CA) behind the name. <u>If the Corresponding Author has changed, please indicate the reason under section 3</u>.

	First name(s)	Family name (this name will appear in full on the final publication and will be searchable in various abstract and indexing databases)	Affiliated institute	E-mail address
1st author	Ru-Huei	Fu	1. Graduate Institute of Biomedical Sciences, China Medical University, Taichung 40402, Taiwan<br>2. Translational Medicine Research Center, China Medical University Hospital, Taichung 40447, Taiwan	rhfu@mail.cmu.edu.tw
2nd author	Po-Yen	Wu	3. Division of Pediatric Neurology, China Medical University Children's Hospital, Taichung 40447, Taiwan	d36088@mail.cmuh.org.tw
3rd author	I-Ching	Chou	3.Division of Pediatric Neurology, China Medical University Children's Hospital, Taichung 40447, Taiwan<br>4.College of Chinese Medicine, Graduate Institute of Integrated Medicine, China Medical University, Taichung 40402, Taiwan	iching@mail.cmuh.org.tw
4th author	Chien-Heng	Lin	5.Division of Pediatrics Pulmonology, China Medical University Children's Hospital, Taichung 40447, Taiwan<br>6.Department of Biomedical Imaging and Radiological Science, College of Medicine, China Medical University, Taichung 40402, Taiwan,	lch227@ms39.hinet.net
5th author	Syuan-Yu	Hong	1.Graduate Institute of Biomedical Sciences, China Medical University, Taichung 40402, Taiwan<br>3.Division of Pediatric Neurology, China Medical University Children's Hospital, Taichung 40447, Taiwan<br>7.Department of Medicine, School of Medicine, China Medical University, Taichung 40402, Taiwan	dazingdog@hotmail.com
6th author				
7th author				
8th author				
9th author				
10th author				

Please use an additional sheet if there are more than 10 authors.

SPRINGER NATURE Springer Nature is one of the world's leading global research, educational and professional publishers, created in May 2015 through the combination of Nature Publishing Group, Palgrave Macmillan, Macmillan Education and Springer Science+Business Media.

SPRINGER NATURE

Change of authorship request form - Journals (pre-acceptance)

Section 5: Author contribution, Acknowledgement and Disclosures. Please use this section to provide a new disclosure statement and, if appropriate, acknowledge any contributors who have been removed as authors and ensure you state what contribution any new authors made (if applicable per the journal or book (series) policy). Please ensure these are updated in your manuscript - after approval of the change(s) - as our production department will not transfer the information in this form to your manuscript.

New acknowledgements:

On behalf of all the authors (the names are as follows: SYH, RHF, PYW, ICC and CHL), we extend our utmost gratitude to the medical and financial support of China Medical University Hospital and China Medical University Children's Hospital (DMR-111-250) to the completion of this article. We express our sincere appreciation to Dr. Li Yingxuan from the Department of Imaging Medicine at China Medical University Hospital, Dr. Wang Zhongxing from the Department of Genetics at China Medical University Hospital. Their invaluable insights and timely guidance have been instrumental in resolving intricate diagnostic cases and facilitating the interpretation of medical images. It is unequivocally due to their invaluable assistance that this article has reached its successful culmination.

New Disclosures (financial and non-financial interests, funding):

Not applicable for financial, non-financial interests or fundings

New Author Contributions statement (if applicable per the journal policy):

SYH provided treatment to the patient, collected the data and wrote the draft. RHF and PYW participated in the design of the study and wrote the manuscript. ICC and CHL provided their experience for the patient' s collection and modified the manuscript accordingly. All authors read and approved the final manuscript.

State 'Not applicable' if there are no new authors.

SPRINGER NATURE Springer Nature is one of the world's leading global research, educational and professional publishers, created in May 2015 through the combination of Nature Publishing Group, Palgrave Macmillan, Macmillan Education and Springer Science+Business Media.

SPRINGER NATURE

Change of authorship request form - Journals (pre-acceptance)

Section 6: Declaration of agreement. *All* authors, unchanged, new and removed *must* sign this declaration.
(NB: Please print the form, (docu)-sign and return/upload a scanned copy. Please note that signatures that have been inserted as an image file are acceptable as long as it is handwritten. Typed names in the signature box are unacceptable.) *** Please delete as appropriate. Delete all of the bold if you were on the original authorship list and are remaining as an author.**

	First name	Family name		Signature	Date
1st author	Ru-Huei	Fu	I agree to the proposed new authorship shown in section 4 **/and the addition/removal*of my name to the authorship list** /and the proposed change in corresponding author	Ru-Huei Fu	August 24, 2023
2nd author	Po-Yen	Wu	I agree to the proposed new authorship shown in section 4 **/and the addition/removal*of my name to the authorship list** /and the proposed change in corresponding author	Po Yen Wu	August 24, 2023
3rd author	I-Ching	Chou	I agree to the proposed new authorship shown in section 4 **/and the addition/removal*of my name to the authorship list** /and the proposed change in corresponding author	I Ching Chou	August 24, 2023
4th authors	Chien-Heng	Lin	I agree to the proposed new authorship shown in section 4 **/and the addition/removal*of my name to the authorship list** /and the proposed change in corresponding author	Chien-Heng Lin	August 24, 2023
5th author	Syuan-Yu	Hong	I agree to the proposed new authorship shown in section 4 **/and the addition/removal*of my name to the authorship list** /and the proposed change in corresponding author	Syuan-Yu Hong	August 24, 2023
6th author			I agree to the proposed new authorship shown in section 4 **/and the addition/removal*of my name to the authorship list** /and the proposed change in corresponding author		
7th author			I agree to the proposed new authorship shown in section 4 **/and the addition/removal*of my name to the authorship list** /and the proposed change in corresponding author		

SPRINGER NATURE Springer Nature is one of the world's leading global research, educational and professional publishers, created in May 2015 through the combination of Nature Publishing Group, Palgrave Macmillan, Macmillan Education and Springer Science+Business Media.

SPRINGER NATURE

Change of authorship request form - Journals (pre-acceptance)

	First name	Family name		Signature	Date
8th author			I agree to the proposed new authorship shown in section 4 /**and the addition/removal*of my name to the authorship list** /and the proposed change in corresponding author		
9th author			I agree to the proposed new authorship shown in section 4 /**and the addition/removal*of my name to the authorship list** /and the proposed change in corresponding author		
10th author			I agree to the proposed new authorship shown in section 4 /**and the addition/removal*of my name to the authorship list** /and the proposed change in corresponding author		

Please use an additional sheet if there are more than 10 authors.

In case of author collaborations with formal agreement:

	Name of consortium/consortia	First name	Family name		Signature	Date
Representative/legal guarantor				I agree to the proposed new authorship shown in section 4 /**and the addition/removal*of my name to the authorship list** /and the proposed change in corresponding author		

Both added/removed authors should complete the information in the first table under Section 6.

---- End of form ----

SPRINGER NATURE Springer Nature is one of the world's leading global research, educational and professional publishers, created in May 2015 through the combination of Nature Publishing Group, Palgrave Macmillan, Macmillan Education and Springer Science+Business Media.
