# Supplementary material for: Demographic and clinical characteristics, seizure disorders, and antiepileptic drug usage in different types of corpus callosum disorders: a comparative study in children
Source: Ital J Pediatr. 2024 Jan 25;50:20. doi: 10.1186/s13052-024-01589-x (PMC10809518; doi:10.1186/s13052-024-01589-x)
Supplement: Supplementary file 2 — Supplementary Material 2 [file 13052_2024_1589_MOESM2_ESM.docx]

**Reviewer reports:**

Reviewer #3: This study provided insights into the demographic and clinical characteristics, seizure disorders, and antiepileptic drug usage among individuals with different types of corpuses callosum disorders. There were some points that should be specified furtherly:

1. The diagnostic basis and methods of different seizure patterns did not be explained in material and methods.

**[Reply]:** The "Materials and Methods" section was revisited to incorporate pertinent information.

2. This article discussed two parts of corpuses callosum, the clinical characteristics and the epilepsy. This article focused on the content related to epilepsy but did not focus on the relationship between the two parts, so the theme was not clear

**[Reply]** A comprehensive revision of the "Discussion" section was undertaken, encompassing a structural overhaul, which notably encompassed an analysis of the interplay between clinical characteristics and epilepsy in the context of corpus callosum disorders (CCD).

3. The genes related to corpus callosum disorders were introduced in the Introduction, but were not mentioned in the following, which was slightly redundant.

**[Reply]** Superfluous details pertaining to genetic factors of CCD were excised from the "Introduction" section, enhancing the overall conciseness of the content.

“Moreover, genetic factors have been implicated in the pathogenesis of corpus callosum disorders. Mutations in genes such as EMX1, EMX2, and L1CAM have been associated with agenesis of the corpus callosum [7,8]. Similarly, environmental factors, including prenatal exposure to teratogenic agents, have been linked to corpus callosum abnormalities [9].” Has been changed to

“**Moreover, genetic and environmental factors, including prenatal exposure to teratogenic agents, have been linked to corpus callosum abnormalities [7-9].**”

4. The statistics of MCD and epilepsy in the results did not reflect the relationship between them well and were not reflected in the conclusion and discussion.

**[Reply]** An additional section was introduced within the "Discussion," denominated "**Distinctive Prevalence of MCD, Diverse Clinical Implications of Various CCD Subtypes, and the Interrelationship Between MCD and Seizure Frequency.**" Concurrently, corresponding modifications were effected within both the "Discussion" and "Conclusions" sections.

5. The article listed the use of antiepileptic drugs in each group but did not mention the efficacy.

**[Reply]** This study rigorously presented a comprehensive exploration of "Demographic and Clinical Characteristics, Seizure Disorders, and Antiepileptic Drug Utilization in Varied Corpus Callosum Disorders." It is imperative to underscore that the central focus of this study does not lie in assessing the efficacy of individual antiepileptic medications or their combinations. Your input will be duly considered in our forthcoming study designs.

Reviewer #4:

1. The article Demographic and Clinical Characteristics, Seizure Disorders, and Antiepileptic Drug Usage in Different Types of Corpus Callosum Disorders: A Comparative Study in Children" has some points of interest in particularly the genetic etiology of Corpus Callosum Disorders (CCD). The Authors should include the genetic etiology on method section and add "West syndrome treatment: New roads for an old syndrome" Pavone Frontiers in Neurology 2013 that focusedA commentary on West syndrome: response to valproate.

**[Reply] As this study adopts a retrospective research approach, the investigation of genetic etiology falls beyond its scope. Nonetheless, we appreciate your recommendation and intend to incorporate the suggested reference (ref. [7]) in our introduction section.**
