# Supplementary material for: Demographic and clinical characteristics, seizure disorders, and antiepileptic drug usage in different types of corpus callosum disorders: a comparative study in children
Source: Ital J Pediatr. 2024 Jan 25;50:20. doi: 10.1186/s13052-024-01589-x (PMC10809518; doi:10.1186/s13052-024-01589-x)
Supplement: Supplementary file 3 — Supplementary Material 3 [file 13052_2024_1589_MOESM3_ESM.docx]

Subject: Submission of Manuscript - "Demographic and Clinical Characteristics, Seizure Disorders, and Antiepileptic Drug Usage in Different Types of Corpus Callosum Disorders: A Comparative Study in Children"

Dear Editor-in-Chief,

I am writing to submit my manuscript entitled "Demographic and Clinical Characteristics, Seizure Disorders, and Antiepileptic Drug Usage in Different Types of Corpus Callosum Disorders: A Comparative Study in Children" for consideration for publication in ***Italian Journal of Pediatrics***.

I believe that this manuscript will make a valuable contribution to the field of epilepsy and related disorders, specifically in children with corpus callosum disorders. The study aims to investigate the demographic and clinical characteristics of children with various types of corpus callosum disorders, focusing on the presence of seizure disorders and the utilization of antiepileptic drugs. By conducting a comparative analysis, we aim to explore potential associations between specific corpus callosum abnormalities, seizure types, and antiepileptic drug effectiveness in this population.

The research involved a well-defined cohort of children with corpus callosum disorders, and we collected comprehensive demographic and clinical data to ensure robust statistical analysis. The study employed rigorous methodologies, including standardized diagnostic criteria and validated assessment tools, to provide reliable and accurate results. We believe that our findings have the potential to enhance our understanding of the relationship between corpus callosum disorders, seizure disorders, and the management of epilepsy in children.

The significance of this study lies in its potential to guide clinicians in the diagnosis and treatment of children with corpus callosum disorders and associated seizure disorders. Furthermore, it has the potential to influence future research directions and contribute to the development of tailored therapeutic strategies in this vulnerable population.

The manuscript has not been published previously and is not under consideration for publication elsewhere. All authors have reviewed the manuscript and have agreed to its submission to **Italian Journal of Pediatrics**

We kindly request that the manuscript be considered for publication in Italian Journal of Pediatrics. We believe that the journal's readership, which comprises researchers, clinicians, and healthcare professionals involved in epilepsy management, would greatly benefit from the insights and implications of our study.

Thank you for considering our manuscript for publication in Italian Journal of Pediatrics. We look forward to hearing from you regarding the outcome of the review process.

Yours sincerely,

**^✽^Correspondence to:** Syuan-Yu Hong (S.-Y. Hong), Department of Pediatrics, China Medical University Hospital, 2 Yuh-Der Road, Taichung 404, Taiwan.

Tel.: +8864-24721087

E-mail addresses: (S.-Y. Hong): **dazingdog@hotmail.com**
